# The AP2/ERF Transcription Factor DRNL Modulates Gynoecium Development and Affects Its Response to Cytokinin

**DOI:** 10.3389/fpls.2017.01841

**Published:** 2017-10-26

**Authors:** Yolanda Durán-Medina, Joanna Serwatowska, J. Irepan Reyes-Olalde, Stefan de Folter, Nayelli Marsch-Martínez

**Affiliations:** ^1^Laboratorio de Identidad Celular de Plantas, Departamento de Biotecnología y Bioquímica, Unidad Irapuato, Centro de Investigación y de Estudios Avanzados del Instituto Politécnico Nacional, Irapuato, Mexico; ^2^Laboratorio Nacional de Genómica para la Biodiversidad, Unidad de Genómica Avanzada, Centro de Investigación y de Estudios Avanzados del Instituto Politécnico Nacional, Irapuato, Mexico

**Keywords:** cytokinins, DRNL/ESR2/BOL, Arabidopsis gynoecium, AHP6, plant development, plant hormones, carpel development, organ development

## Abstract

The gynoecium is the female reproductive system in flowering plants. It is a complex structure formed by different tissues, some that are essential for reproduction and others that facilitate the fertilization process and nurture and protect the developing seeds. The coordinated development of these different tissues during the formation of the gynoecium is important for reproductive success. Both hormones and genetic regulators guide the development of the different tissues. Auxin and cytokinin in particular have been found to play important roles in this process. On the other hand, the AP2/ERF2 transcription factor BOL/DRNL/ESR2/SOB is expressed at very early stages of aerial organ formation and has been proposed to be a marker for organ founder cells. In this work, we found that this gene is also expressed at later stages during gynoecium development, particularly at the lateral regions (the region related to the valves of the ovary). The loss of *DRNL* function affects gynoecium development. Some of the mutant phenotypes present similarities to those observed in plants treated with exogenous cytokinins, and *AHP6* has been previously proposed to be a target of DRNL. Therefore, we explored the response of *drnl-2* developing gynoecia to cytokinins, and found that the loss of *DRNL* function affects the response of the gynoecium to exogenously applied cytokinins in a developmental-stage-dependent manner. In summary, this gene participates during gynoecium development, possibly through the dynamic modulation of cytokinin homeostasis and response.

## Introduction

In contrast to many animals, plants can make new organs postembryonically. Stem cells produce signals to maintain a certain group of cells in an undifferentiated state with active cell division, which we call a meristem (reviewed in Gaillochet and Lohmann, 2015). Cells on the periphery of shoot and flower meristems obtain the capacity to differentiate and will develop into lateral organs. Thereby, plant growth is maintained (reviewed in Hepworth and Pautot, 2015; Taylor-Teeples et al., 2016), which may last over thousands of years in the case of, for example, the immense Sequoia trees.

The formation of new organs goes mostly together with the presence of the hormone auxin, observed in only one or just a few cells just before organ primordium emergence, which are called the organ founder cells (Aloni et al., 2003, 2006; Reinhardt et al., 2003; Chandler et al., 2011b). *BOL/DRNL/ESR2/SOB* (*BOLITA, DÖRNROSCHEN-LIKE, ENHANCER OF SHOOT REGENERATION 2, SUPPRESSOR OF PHYTOCROME B*) is an AP2/ERF transcription factor that functions at early stages of organogenesis (Ikeda et al., 2006; Marsch-Martinez et al., 2006; Ward et al., 2006; Chandler et al., 2007). It has been attributed several functions that have arisen mainly from the observed phenotypes of overexpression (*35S::ESR2-ER*) and gain or loss of function of this gene (*bol-D* and *drnl-2*) (Ikeda et al., 2006; Marsch-Martinez et al., 2006; Chandler et al., 2007; Nag et al., 2007). It is expressed at very early stages of aerial organ formation, and its overexpression induces the formation of ectopic organs in tobacco, and the formation of green calli in Arabidopsis roots that can form leaves, inflorescences and flowers when detached from the root (Ikeda et al., 2006; Marsch-Martinez et al., 2006). This indicates that this transcription factor is able to promote organ formation. Its expression has been characterized in detail at the earliest stages of organ formation, and it has been proposed that this gene is a marker for the flower organ founder cells in Arabidopsis (Chandler et al., 2011b). The loss of *DRNL* and *DRN/ESR1* (*DÖRNROSCHEN*/*ENHANCER OF SHOOT REGENERATION 1*, its closest homolog) function in Arabidopsis causes cotyledon fusions, though this phenotype does not present full penetrance (Chandler et al., 2007). Moreover, the loss of *DRNL* function also causes diverse alterations in the organs of all floral whorls (Nag et al., 2007). In the reproductive organs of the flower, these alterations are very severe in the stamens and have been well characterized. Gynoecium phenotypes have been reported to be less severe, being mostly normal but occasionally misshapen and bent (Nag et al., 2007). The valves of *drnl-2* gynoecia can be absent or asymmetric, though these defects occur at low penetrance (6%, Chandler et al., 2011b), while gynoecia of the triple *drnl drn puchi* (*PUCHI* is a third close homolog; Hirota et al., 2007; Karim et al., 2009) mutant do not develop valves (Eklund et al., 2011).

**FIGURE 1:**
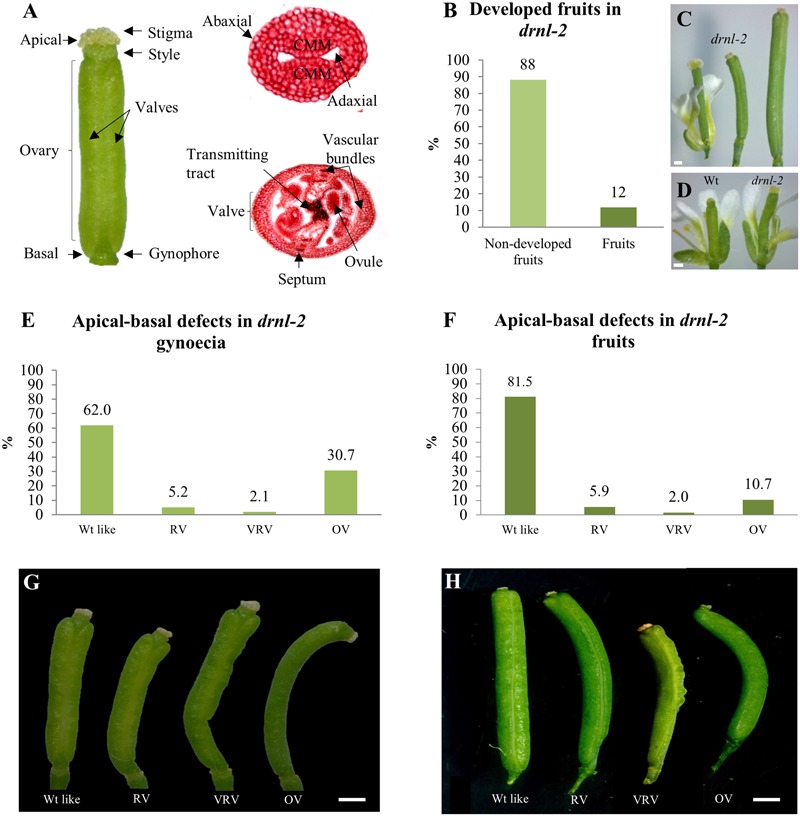
External defects in *drnl-2* mature gynoecia and fruits. **(A)** Structures of the Arabidopsis gynoecium. **(B)** Proportion of gynoecia that become fruits in *drnl-2* plants. **(C)** *drnl-2* flower (left), a *drnl-2* gynoecium that did not develop into fruit (middle), and a *drnl-2* developing fruit (right). **(D)** Comparison between wild type (WT) and *drnl-2* flowers at stage 13. **(E,G)** Apical–basal defects in *drnl-2* gynoecia, frequencies **(E)** and phenotypes **(G)**. **(F,H)** Apical–basal defects in *drnl-2* fruits, frequencies **(F)** and phenotypes **(H)**. Scale bars: 0.2 mm in **(C)** and **(D)**; 0.5 mm in **(G)**; 1 mm in **(H)**.

The pistil or gynoecium is a very important part of the flower, because it is the female reproductive system that will give rise to the fruit at a later stage of development (reviewed by Roeder and Yanofsky, 2006; Alvarez-Buylla et al., 2010). Like most angiosperms, in Arabidopsis each flower produces a gynoecium in the center. The gynoecium consists of different structures, and, in the apical–basal axis (**Figure 1A**), at the top it has a stigma with the style below, then the ovary with valves that protect the ovules, and finally the gynophore at the bottom. In Arabidopsis, the ovary is formed by two fused carpels. The floral meristem gives rise to the carpel primordia, and two congenitally fused carpels will arise and form a kind of hollow tube that during development will close at the top, followed by differentiation at the apical end, where the style and stigma will be formed. Inside the hollow tube, two meristematic regions will be formed along the side where the carpels are fused; these regions are also called the carpel margin meristem (CMM). The CMM gives rise to all the internal tissues, septum, placenta, ovules, transmitting tract, tissues that are crucial for the reproductive competence of the plant (reviewed in Bowman et al., 1999; Alvarez and Smyth, 2002; Wynn et al., 2011; Reyes-Olalde et al., 2013). As expected, transcription factors are essential for correct gynoecium development (reviewed in Alvarez-Buylla et al., 2010; Ferrándiz et al., 2010; Reyes-Olalde et al., 2013; Chávez Montes et al., 2015), but also hormones like auxin and cytokinin are important for its proper patterning and morphogenesis (reviewed in Sehra and Franks, 2015; Marsch-Martinez and de Folter, 2016). The pathways of these two hormones are connected at different levels. Both hormones are well studied, and antagonistic but also synergistic functions have been described for different tissues and organs (reviewed by El-Showk et al., 2013; Schaller et al., 2015). For instance, alterations in auxin signaling or biosynthesis, application of auxin, or inhibitors of auxin transport, affect the apical–basal axis of the gynoecium, i.e., the proportion of organ sizes relative to each other is affected along this axis. One of these alterations is that valves grow at different sizes (asymmetric valves; Sessions and Zambryski, 1995; Nemhauser et al., 2000; Cheng et al., 2006; Sohlberg et al., 2006; Stepanova et al., 2008; Zuñiga-Mayo et al., 2014). The same effects are observed when exogenous cytokinin is applied (Zuñiga-Mayo et al., 2014). Interestingly, asymmetric valves have also been reported for the *drnl* mutant (Chandler et al., 2011b), suggesting that *DRNL* also plays a role during gynoecium development, probably by affecting hormonal pathways. Therefore, we characterized *DRNL* function during gynoecium development by studying the effects of its loss of function, following its expression during development, and exploring its connection with the phytohormone cytokinin.

## Materials and Methods

### Plant Materials and Growth Conditions

The lines used in this study were wild type (WT) ecotypes Landsberg *erecta* (L*er*) and Columbia (Col); mutants *bol-D* (Marsch-Martinez et al., 2006), *drnl-2* (Nag et al., 2007), *ahp6-1* (Besnard et al., 2014); reporter lines *BOL::GUS* (comprising 1550 nucleotides upstream the start codon; Marsch-Martinez et al., 2006), *AHP6*::*GFP* (comprising 1594 nucleotides upstream the start codon; Mähönen et al., 2006), and the inducible *DRNL* line *35S::DRNL-ER* (Ikeda et al., 2006; Eklund et al., 2011).

All genotypes were germinated in soil (peat moss, perlite and vermiculite 3:1:1) under long-day conditions (16–8 h, light–dark) in a growth chamber at 22°C. Two weeks after germination, the plants were transferred to a greenhouse with a temperature range from 22 to 28°C, and natural light conditions. Day length varied in different seasons.

### Gynoecium Phenotypic Analyses

Fruits were evaluated in *drnl-2* and L*er* plants, which were germinated and grown under the same conditions as in the rest of the experiments. The numbers of fruits (siliques) and pistils that did not develop into fruits per plant were registered (*n* = 14 plants). Fruits were collected and classified according to their phenotype (*n* = 205 fruits). For the phenotypic analysis of pistils that did not develop into fruits, 199 pistils present along inflorescence stems were analyzed. Images were captured using a Stemi 2000-C microscope (Carl Zeiss, Oberkochen, Germany).

### *BOL::GUS* Expression

β-Glucuronidase staining was performed for 24–168 h at 37°C in a 2 mM X-Gluc solution (Gold Biotechnology), using established protocols (Campisi et al., 1999). *BOL* expression was observed under a DM6000B microscope coupled with a DFC420 C camera (both from Leica).

### *AHP6::GFP* Expression

To analyze the regulation of *AHP6* expression in response to BOL activity, one drop of β-estradiol or mock solution was applied per inflorescence in *DRNL-ER AHP6::GFP* plants. The β-estradiol solution contained 10 μM β-estradiol (Sigma–Aldrich) with 0.01% Silwet L-77 (Lehle Seeds). A solution containing DMSO and Silwet L-77 in the same concentration as in the β-estradiol solution was used for the mock treatment.

Transverse sections of the gynoecia were made 48 h after the treatments, according to Reyes-Olalde et al. (2013). The sections were visualized and images were captured using a LSM 510 META confocal scanning laser inverted microscope (Carl Zeiss). Propidium Iodide (PI; at 0.01 mg/mL) was used as a counterstain. PI was excited using a 514-nm line and GFP was excited using a 488-nm line of an Argon laser. PI emission was filtered with a 575-nm long pass (LP) filter and GFP emission was filtered with a 500–550-nm bandpass (BP) filter.

### Histological Sections

Tissues were fixed in FAE (3.7% formaldehyde, 5% glacial acetic acid, and 50% ethanol) with vacuum (20 min, 4°C) and incubated for 120 min at room temperature. The material was rinsed with 70% ethanol and incubated overnight at 4°C, followed by dehydration in a series of ethanol solutions (85, 95, and 100%) for 60 min. each and embedded in Technovit^®^ 7100 according to the manufacturer’s instructions (Heraeus-Kulzer, Wehrheim, Germany). Using a rotary microtome (Reichert-Jung 2040; Leica), 10 μm thick transverse sections of L*er* and *drnl-2* inflorescences were made and stained with alcian blue (0.5% pH 3.1; Sigma–Aldrich) for 25 min and neutral red (0.5%) for 5 min. Micrographs were obtained using a DM6000B microscope coupled with a DFC420 C camera (both from Leica).

### Gene Expression Analysis

For qRT-PCR analysis, open flowers were removed, inflorescences with only floral buds were collected, and total RNA was extracted using the Quick-RNA^TM^ MiniPrep Kit (Zymo Research). The samples were treated with DNase I, included in the kit. Reverse transcription and amplification were performed using a KAPA SYBR FAST One-Step qRT-PCR Kit (Sigma–Aldrich) in a StepOne^TM^ thermocycler (Applied Biosystems). Three biological replicates and three technical replicates were included in the analysis. Target gene expression levels were normalized to *ACTIN 2*. Data was analyzed using the 2^-ΔΔC_T_^ method (Livak and Schmittgen, 2001). In the graphs, each bar (1, 2, and 3) represents one biological replicate, and error bars represent the standard error corresponding to three technical replicates of each biological replicate.

### Cytokinin Treatments

Cytokinin treatments were performed in a similar way as described by Zuñiga-Mayo et al. (2014). The experiment was carried out in greenhouse conditions with natural light in autumn, and all plants (L*er* and Col as WT, *drnl-2* and *ahp6*) were grown simultaneously under the same conditions. When the first fruits were observed in the inflorescence stem, those fruits were removed, leaving only closed buds in the inflorescences. Once this was done, BAP solution drops (100 μM 6-benzylaminopurine; Duchefa Biochemie, Haarlem, Netherlands) and 0.01% Silwet L-77 (in distilled water) were applied on the inflorescences for five consecutive days. Sixteen days after treatment the gynoecia were collected and analyzed in chronological order of development. The mock solution contained Silwet L-77 and the same concentration of NaOH (0.2 mN) used to prepare the hormone solution.

## Results

### Apical–Basal Defects in *drnl-2* Mutant Gynoecia

To obtain information about the role of *DRNL* during gynoecium development, *drnl-2* (Nag et al., 2007) mutant gynoecia were compared to WT gynoecia. *drnl-2* gynoecia were slightly longer and presented a broader stigma than the L*er* WT gynoecia (**Figure 1D**). This mutant presents reduced fertility (Nag et al., 2007), and a large proportion of gynoecia do not develop as fruits. Instead of elongating as a normal fertilized gynoecium converted into a developing fruit, they maintain the size of a gynoecium at stage 13 (**Figure 1C**; middle). Only about 12% of *drnl-2* gynoecia developed into a fruit in our growth conditions (**Figure 1B**), in comparison to WT plants, where most gynoecia are fertilized and become fruits. Out of this 12% of gynoecia that converted into fruits, different altered phenotypes along the apical–basal axis of the fruits were observed, and were most evident in the ovary region. These phenotypes were classified in 4 types: WT-like, reduced valves (RV), very reduced valves (VRV), and one valve (OV) (**Figure 1H**). The 81.5% of the total number of fruits presented a WT-like phenotype. These fruits had symmetrical valves like those of WT plants. The rest of the fruits presented defects in the symmetry of the valves: 5.9% had reduced valves, 2% had very reduced valves, and interestingly, the percentage of fruits with only one valve was greater than that of fruits with asymmetric valves (10.7%) (**Figure 1F**).

Besides fruits, we also analyzed the phenotype of the gynoecia that stayed in the stem but did not develop as fruits (**Figure 1C**, middle). These structures also presented equivalent phenotypes as those observed in developed fruits, but the frequency of the one-valve phenotype was higher (**Figures 1E,G**). The 61.98% of these pistils presented a WT phenotype, 5.21% had reduced valves, 2.08% very reduced valves, and 30.73% presented the one-valve phenotype.

Another phenotype that was observed in *drnl-2* fruits and gynoecia, was that some were curved, a characteristic that had also been mentioned by Nag et al. (2007). This curvature was more marked in gynoecia that presented the “one valve” phenotype (**Figures 1G,H**). On the other hand, interestingly, in some of the gynoecia or fruits presenting “one valve,” this “valve” had almost the width of two valves. While we could clearly distinguish the replum and margins of the valve on one side of the fruit or gynoecium, these structures were not visible on the opposite side. In some fruits and gynoecia it was possible to partially distinguish the presence of these structures in some regions, and appeared to be absent in others (Supplementary Figure 1). In summary, *drnl-2* fruits presented evident external phenotypic alterations, mostly at the valves, at partial penetrance.

### The Loss of *DRNL* Function Causes Defects at Different Stages during Gynoecium Development

**FIGURE 2:**
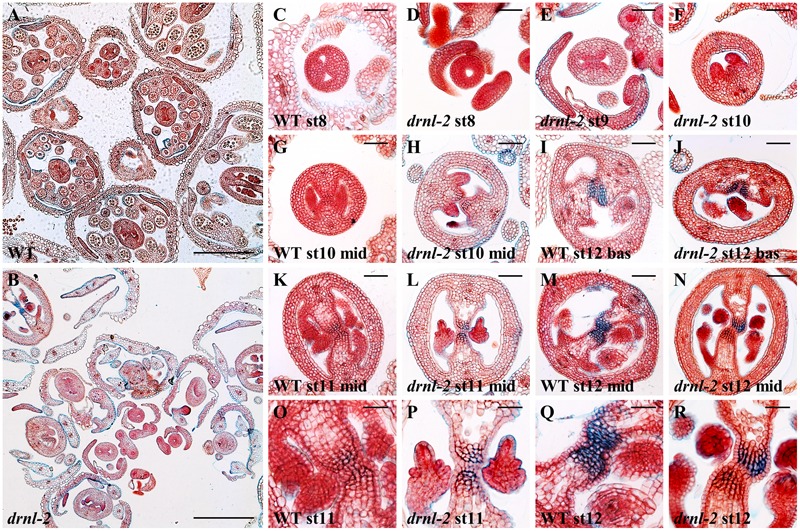
Cross sections of developing WT and *drnl-2* inflorescences and gynoecia. **(A,B)** Cross-sections through WT (L*er*) **(A)** and *drnl-2* **(B)** inflorescences. Valve asymmetries and differences in bud development can be observed. **(C–N)** Whole gynoecia at different stages of development. Characteristic WT early gynoecium **(C)**, where the two internal ridges form a “bowtie” shape. **(D)** Example of an early *drnl-2* gynoecium where the two ridges are not present and a circular aperture is visible. **(E,F)** Examples of *drnl-2* asymmetric valve development at intermediate stages, sometimes presenting a single valve **(F)**. Example of a fully developed *drnl-2* gynoecium with valve asymmetry at its basal region **(J)** compared to the equivalent region in a WT gynoecium **(I)**. **(H,L,N)** Examples of *drnl-2* developing gynoecia with normal valve symmetry at their middle region, compared to WT gynoecia **(G,K,M)**. (**L** and **N**, magnified in **P** and **R**) Examples of asynchrony in the development of the internal tissues of almost mature *drnl-2* gynoecia at stages 11 and 12, compared to their equivalent WT counterparts (**K** and **M**, magnified in **O** and **Q**). Bars: 25 μm in **(O–R)**, 50 μm in all other panels.

After observing the external phenotypes of the *drnl-2* fruits, we also examined the internal tissues of developing gynoecia, and compared them to WT gynoecia. Histological sections allowed the visualization of the different tissues of *drnl-2* gynoecia during development. **Figures 2A,B** present sections of whole WT and *drnl-2* inflorescences, where differences in bud development and some valve asymmetries can be observed, and are shown in more detail in **Figures 2D–F,J**. The valve asymmetries were related to the ones observed in *drnl-2* “mature” gynoecia and fruits (in total, in between 40 and 20% of them presenting defects, respectively, **Figures 1E,F**). As observed in those gynoecia and fruits, the defects observed in *drnl-2* developing gynoecia, do not appear to be 100% penetrant, and also vary in severity from pistil to pistil. Examples of altered growth were present from early gynoecium developmental stages. In **Figure 2D**, a doughnut-shaped, possibly stage 8 young *drnl-2* gynoecium can be observed, presenting a roundish central opening. In contrast, stage 8 (according to Smyth et al., 1990) WT gynoecia normally have an oval shape, present a bowtie shaped central opening, and the valve region can be clearly identified (**Figure 2C**). Examples of valve asymmetrical growth in stage 9 and stage 10 *drnl-2* are presented in **Figures 2E,F**. The image in **Figure 2F** possibly corresponds to the “one valve” phenotype presented in **Figures 1G,H**), because the same structure was observed along the apical–basal axis of the gynoecium in different sections. Developmentally retarded and deformed *drnl-2* stamens, were also observed, as previously described (Nag et al., 2007). Alterations in the valves were more evident at the basal regions of the mutant fruits and gynoecia (Supplementary Figure 1) compared to the WT. **Figure 2J** presents an example of a fully developed gynoecium showing asymmetric valve growth at its basal region (compared to an equivalent region of a WT gynoecium in **Figure 2I**). On the other hand, as observed in fruits and old gynoecia, many developing gynoecia presented normal valve symmetry at their middle region (**Figures 2H,L,N**, compared to WT **Figures 2G,K,M**). Some gynoecia also presented uneven development of their inner tissues, and examples are shown in **Figures 2L,N,P,R**, compared to their equivalent WT counterparts in **Figures 2K,M,O,Q**. **Figure 2P** (a magnification of the center of the image in **Figure 2L**) shows an example of a *drnl-2* ovary where the septum presents cell death and the transmitting tract presents the characteristic staining with alcian blue of a gynoecium at late developmental stages. However, ovule development seems to be delayed since the integuments of the ovules have not yet grown to enclose the female gametophyte, as occurs in the WT at this point (**Figure 2O**). **Figure 2R** (a magnification of **Figure 2N**) shows the transmitting tract region of an ovary where one side presents the characteristic blue staining and cell death (observed as an empty space in the septum), but the other not, which is uncommon in WT ovaries (**Figures 2I,M,Q**). In summary, valve defects can be detected in *drnl-2* gynoecia throughout development, and some gynoecia also present asymmetric or asynchronic development of other tissues.

### *DRNL* Is Expressed at the Prospective Valves of the Gynoecium

Since *drnl-2* mutants presented clear alterations in gynoecium morphology at different stages during gynoecium development, we analyzed *DRNL* expression throughout this process to know whether it was also expressed at intermediate stages of development.

**FIGURE 3:**
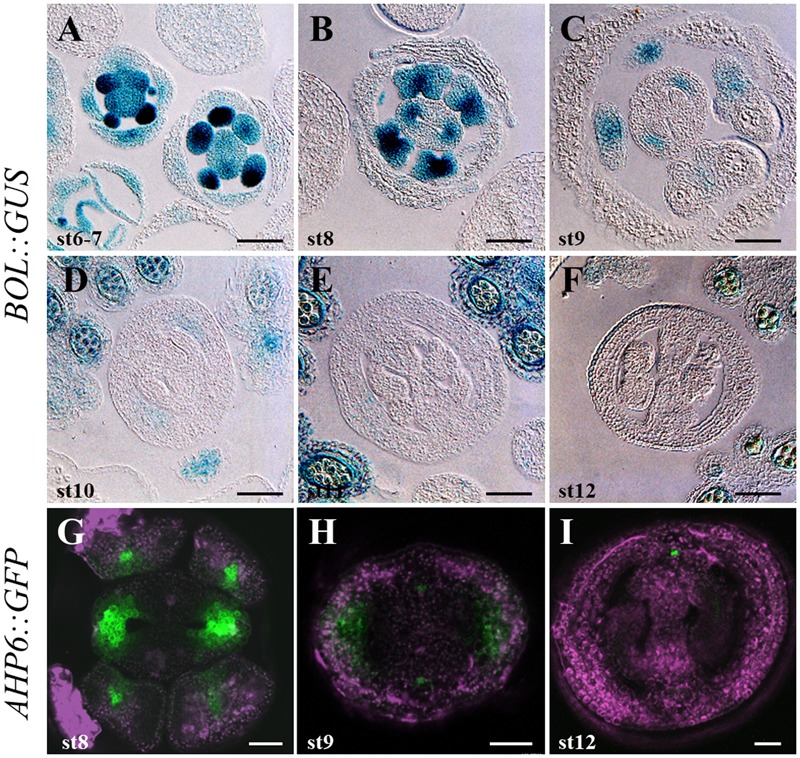
*DRNL/BOL* and *AHP6* expression during gynoecium development. **(A–F)** *DRNL/BOL* expression pattern in inflorescence transverse sections. During floral stages 6–7, the *BOL::GUS* marker expression can be detected in stamen and gynoecium primordia, slightly enhanced at the lateral region of the gynoecium primordium **(A)**. By stage 8, *GUS* staining becomes restricted to the developing valves, except for their epidermal layer **(B)**. During stages 9–11 **(C–E)**, *BOL* expression is found at the valves, becoming weaker and restricted to the adaxial cell layers. *GUS* activity is also found in stamens **(C)** and, after that, all over the anther **(D,E)**. By stage 12, *BOL* expression is no longer detected in the gynoecium, but is detectable in microspores and anther tapetum **(F)**. **(G–I)** *AHP6* expression pattern in inflorescence transverse sections. In the gynoecium, *AHP6* is similarly expressed from early developmental stages on. Similar expression in the valves at stage 8 **(G)** and early 9 **(G)** is shown. As *DRNL/BOL, AHP6* is not detected anymore in the valves of stage 12 gynoecia **(I)**. Scale bars: 50 μm in **(A–F)**; 20 μm in **(G–I)**.

To determine the expression pattern of *DRNL* in the reproductive tissues of developing flowers, we performed GUS staining of a *BOL::GUS* line (Marsch-Martinez et al., 2006), which revealed that GUS activity was found in developing gynoecia and stamens through different developmental stages (**Figure 3** and Supplementary Figure 2). During floral stages 6 and 7 (**Figure 3A**; floral stages according to Smyth et al., 1990), prior to the clear differentiation of the inner meristematic outgrowths of the emerging gynoecium (CMMs), *DRNL* expression was found in stamen and gynoecium primordia, as previously reported (Nag et al., 2007; Chandler et al., 2011b). Interestingly, we observed that *DRNL* expression was enhanced in the regions that will later give rise to the valves. By stage 8, when the two CMMs form at the inner medial domain, GUS staining became restricted to the developing valves, at the lateral domains of the gynoecium, but expression was not detected in the epidermal cell layer. At this stage, high *DRNL* expression could also be detected in the early developing stamens (**Figure 3B**). The activity of the *DRNL* promoter was maintained at the valves of developing gynoecia until stage 11 (**Figures 3C–E**), although as development proceeded, it became weaker and more restricted to the adaxial cell layers. At these stages, GUS activity was also found in stamens, mostly at the apical zone and, afterward, throughout the anther (**Figures 3C–E**) as reported by Nag et al. (2007). At anthesis, by stage 12, *DRNL* promoter activity was no longer found in the gynoecium (**Figure 3F**). However, expression was still detectable in the anthers in microspores and the tapetum (**Figure 3F**), as previously reported (Nag et al., 2007).

### DRNL Can Regulate *AHP6* during Gynoecium Development

The observed defects in the *drnl-2* gynoecia resembled to a certain extent, gynoecia of mutants affected in hormone transport, response, or gynoecia that have been treated with hormones or hormone transport inhibitors (Sessions and Zambryski, 1995; Nemhauser et al., 2000; Cheng et al., 2006). Auxin has an important role during gynoecium development, and previous reports have related *DRNL* function to auxin (Chandler, 2008; Eklund et al., 2011), though *DRNL* may be also participating in other processes. Interestingly, exogenous cytokinin treatments are also able to affect the proper establishment of the apical–basal patterning in the Arabidopsis gynoecium (Zuñiga-Mayo et al., 2014). These defects promoted by cytokinin treatments in WT gynoecia resemble some of the alterations observed in *drnl-2* mutant gynoecia (**Figure 1**). This resemblance suggested that there could be a possible relation of *DRNL* with cytokinins during gynoecium development. Therefore, we sought reported genes that participate in the cytokinin pathway and that have been found to be connected to *DRNL*. One of these genes is *AHP6*, which encodes a histidine phosphotransfer protein that negatively modulates cytokinin signaling (Hwang et al., 2002; Mähönen et al., 2006). This gene has been proposed to be a possible DRNL target by Ikeda et al. (2006). Like *DRNL, AHP6* is expressed in the inflorescence meristem in the regions where floral organ primordia develop (Besnard et al., 2014; Supplementary Figure 2A). Recently, *AHP6* was reported to be expressed at the lateral domains of the gynoecium in stages 7–9 (Reyes-Olalde et al., 2017). Therefore, we compared *AHP6* and *DRNL* expression to find out whether they shared similar expression patterns during gynoecium development.

We used an *AHP6::GFP* reporter line (Mähönen et al., 2006) and observed that *AHP6* is expressed at early stages of gynoecium development, in a similar way to *DRNL*. Expression of *AHP6* was observed in the developing flower as two light spots in the prospective gynoecium region (stage 4 of development; Supplementary Figure 2C). *AHP6* expression also marked the region where the valves will develop at later stages and was maintained until stage 7. From stage 8 onward (**Figures 3G,H**), its expression began to decrease in this region, and then at stage 9, a more confined expression of *AHP6* in the valves was observed. *AHP6* expression disappeared in the valves at stage 12, but it remained in the medial vasculature (**Figure 3I**).

These analyses indicated that *AHP6* and *DRNL* have very similar expression patterns. Both are expressed in the prospective valves during early stages of development. Their expression is lost in the lateral domain of the gynoecium at stage 12, when the tissue is mature. In addition, *DRNL* and *AHP6* not only shared expression locations at the gynoecium, but also in other structures such as stamen primordia (**Figure 3** and Supplementary Figure 2).

**FIGURE 4:**
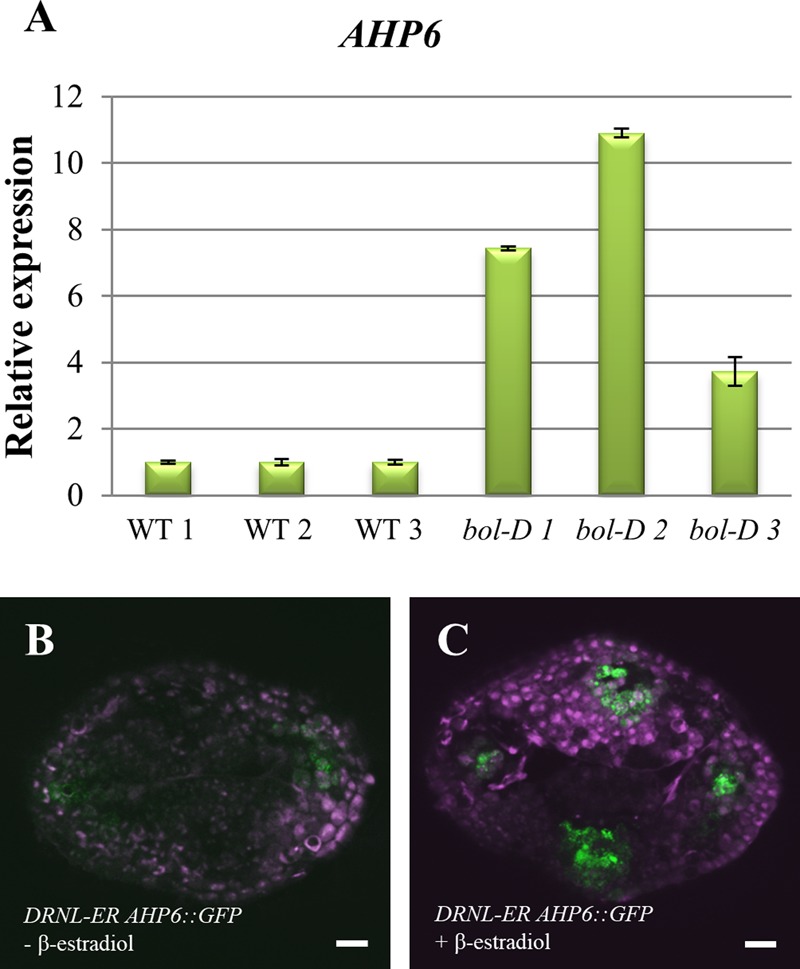
Regulation of *AHP6* expression by DRNL. **(A)** *AHP6* relative expression in *bol-D* mutant inflorescences. Each bar (1, 2, and 3) represents a biological replicate, and standard error bars were calculated from three technical replicates. **(B)** *AHP6* expression in *DRNL-ER AHP6::GFP* (*DRNL-ER* = *35S_pro_::DRNL-ER)* gynoecia at stage 8, treated with mock solution. **(C)** *AHP6* expression in *DRNL-ER AHP6::GFP* gynoecia at stage 8, induced with β-estradiol. Increased *AHP6* expression after induction of DRNL function is observed.

After this similarity in the expression patterns in the gynoecium was observed, we sought to determine whether the gain or loss of *DRNL* function could affect *AHP6* expression. For this, we first analyzed the expression of *AHP6* in the gain of function *bol-D* mutant (a *DRNL* activation tagging allele; Marsch-Martinez et al., 2006). When RNA obtained from whole inflorescences was assayed by qRT-PCR, the accumulation of the *AHP6* transcript was considerably increased in *bol-D*, in comparison to WT inflorescences (**Figure 4A**). We then sought to determine whether this upregulation of *AHP6* expression through DRNL was occurring at the gynoecium. For this, a cross between a DRNL activity inducible line (Ikeda et al., 2006; Eklund et al., 2011) to the *AHP6* reporter line (*DRNL-ER AHP6::GFP*) was performed. We observed that induction of DRNL activity caused a change in the *AHP6* expression pattern in the gynoecium, detectable 48 h after the β-estradiol treatment (**Figures 4B,C**). In stages 8–9 gynoecia, *AHP6* expression was not detected in the provasculature of the medial region, but when DRNL activity was induced we could observe that the expression of *AHP6* appeared in this tissue, and was slightly increased in its normal domain of expression (**Figure 4B**).

Next, we analyzed *AHP6* expression in the *drnl-2* loss of function mutant. qRT-PCR analysis was performed in mutant and WT inflorescences, and no evident decrease in *AHP6* expression in the inflorescences of *drnl-2* could be detected (Supplementary Figure 3). In summary, the increased activity of DRNL is able to upregulate *AHP6*, but DRNL does not appear to be a general regulator of *AHP6* expression in all tissues, possibly regulating it in very specific domains.

### The Loss of *DRNL* Function Alters the Response of the Gynoecium to Cytokinins

The upregulation of *AHP6* by DRNL suggested that DRNL could be modulating cytokinin homeostasis. To test this further, we explored whether the sensitivity of gynoecia to cytokinins was affected by the loss of *DRNL* function. The application of exogenous cytokinins, besides inducing apical–basal defects, also promotes tissue proliferation in the external medial region of the gynoecium (Marsch-Martinez et al., 2012), depending on the developmental stage at which the gynoecium receives the treatment. Because the effect of this treatment in developing gynoecia is very evident, we used it to evaluate the ability of *drnl-2* gynoecia to respond to exogenous cytokinins. We applied cytokinins to *drnl-2* inflorescences and compared the effects of the treatment to WT inflorescences treated in the same way. In parallel, considering that *AHP6* appeared to be regulated by DRNL, we also applied cytokinins to the *ahp6* loss of function mutant, to compare whether its response to the cytokinin application was comparable to the response of *drnl-2*.

For this, inflorescences of *drnl-2, ahp-6*, and their corresponding WT ecotypes were treated once a day for a period of 5 days with a 100 μM BAP solution. All fruits present in the plants were removed before the beginning of the treatment. After 5 days of treatment, the flowers, pistils, and fruits were allowed to develop for 15 days more. After this period, gynoecia were detached from the stem and analyzed in chronological order from the base to the apex of the stem (i.e., from “oldest” to “youngest”).

**FIGURE 5:**
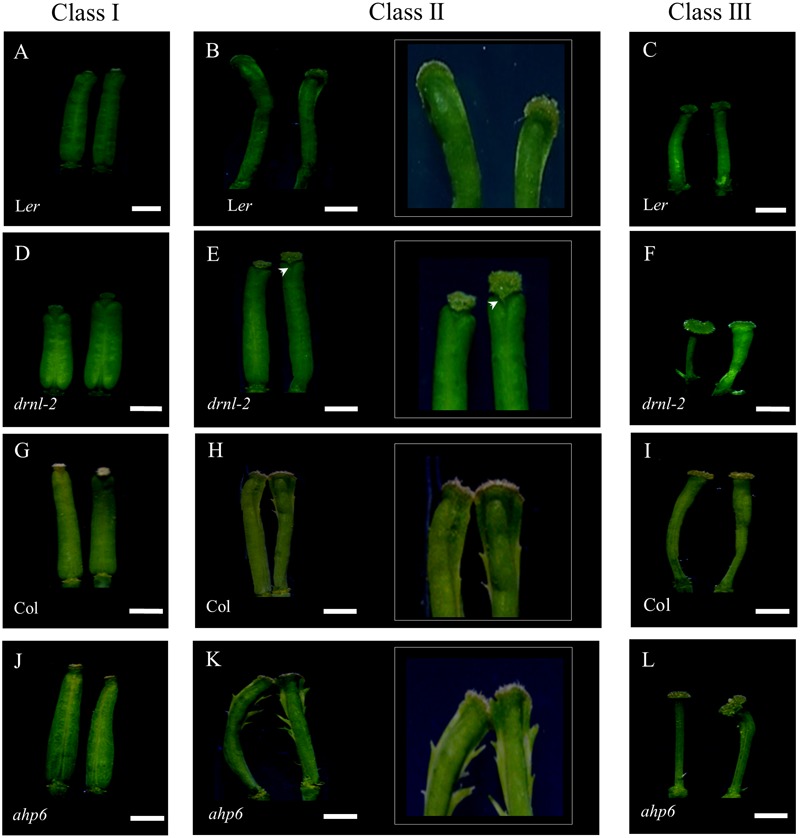
Response of *drnl-2, ahp6*, and WT gynoecia to cytokinin treatment (BAP). **(A–C)** L*er* gynoecia. **(D–F)** *drnl-2* gynoecia. **(G–I)** Col gynoecia. **(J–L)** *ahp6* gynoecia. Gynoecia were classified in three classes according to the developmental stage in which they were when they received the BAP treatment, and the phenotype they presented after the treatment. Class I: gynoecia that were at late developmental stages, with subtle alterations in morphology, class II gynoecia that were at intermediate stages, where tissue proliferated in their external medial region, and class III gynoecia that were at early stages of development, that presented apical–basal defects as a response to BAP. **(B,E,H,K)** show magnifications of the apical region of class II gynoecia to highlight tissue proliferation in the medial region. The arrowhead **(E)** highlights the absence of tissue proliferation in the medial region in comparison to the rest of the ecotypes. Scale bars: 1 mm in the non-magnified images of **(A–L)**.

**FIGURE 6:**
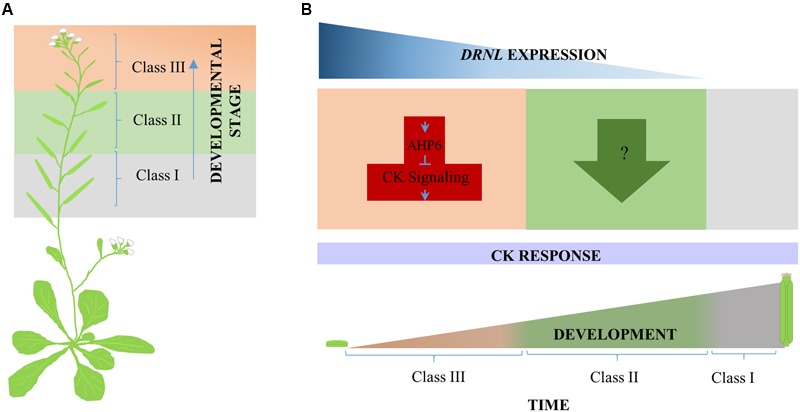
Proposed model for *DRNL* cytokinin response modulation during gynoecium development. **(A)** Three classes of phenotypes were observed along the inflorescence stem in response to cytokinin exogenous application. From bottom to top, Class I, Class II, and Class III. **(B)** According to the different responses of developing *drnl-2* gynoecia to cytokinin, DRNL appears to modulate cytokinin homeostasis (or to participate in the final output of cytokinin signaling) in at least two different ways during development. At very early stages of gynoecium development, DRNL represses the cytokinin response, most likely through the activation of the cytokinin signaling repressor *AHP6*, and maybe other genes, like *CKX7*. At intermediate stages of development, however, the effects of cytokinin application suggest that *DRNL* positively modulates the response to cytokinins through another mechanism (or is required for cytokinin response output). In the developmental time line, orange represents early, green represents intermediate, and gray late gynoecium developmental stages, where *DRNL* is not expressed.

From this experiment, it became evident again that the response to cytokinins depends on the developmental stage in which each gynoecium was at the time of treatment. It should be noted that the treated gynoecia did not continue with their normal development to fruit. These gynoecia were small and showed a gradient of phenotypes as we previously reported (Zuñiga-Mayo et al., 2014) (**Figure 5**). Compared to untreated gynoecia and fruits (Supplementary Figure 4) the observed phenotypes were classified into three categories (**Figures 5, 6A**), according to the developmental stage at which the gynoecia were when they were treated, and the resulting WT phenotype upon cytokinin treatment: Class I, gynoecia that were at late stages of development at the beginning of the treatment (around stages 12–13) (**Figures 5A,D,G,J**), which became short and wide after the treatment; Class II, gynoecia that were at intermediate stages of development (around stages 9–11) (**Figures 5B,E,H,K**), which presented tissue proliferation in their external medial region at the end of the treatment; and Class III, which were at early stages of development (around stages 6–8) at the beginning of the treatment, and presented apical–basal defects at the end of the treatment (**Figures 5C,F,I,L**). Both L*er* and Col WT gynoecia presented these classes of phenotypes, though they were more severe in the Col ecotype.

When *drnl-2* mutant gynoecia were analyzed, it was clear that they presented an altered response to cytokinin. First, *drnl-2* gynoecia equivalent to WT class II gynoecia did not present the evident proliferation of tissue in the external medial region that characterizes the response in WT gynoecia (**Figure 5E**). This lack of overproliferation indicates that these *drnl-2* gynoecia are less responsive to cytokinins. However, younger *drnl-2* gynoecia that were equivalent to class III WT gynoecia, presented the opposite. We detected more severe apical–basal defects than those observed in treated WT gynoecia (**Figure 5F**). These defects were so severe that some *drnl-2* gynoecia did not even develop valves, whereas in WT plants this defect was not observed in the conditions used for this experiment (**Figure 5F**). Therefore, while older *drnl-2* gynoecia presented a reduced response, younger *drnl-2* gynoecia presented an increased response to the cytokinin treatment, compared to WT gynoecia. These results suggest that *drnl-2* gynoecia may be more sensitive or responsive to cytokinins at early stages of development (stages 6–8), whereas at later stages (stages 9–11) mutant gynoecia are less sensitive or responsive to the external application of this phytohormone.

On the other hand, *ahp6* gynoecia appear to be more sensitive to cytokinins than WT gynoecia at the stages analyzed, particularly young gynoecia. Both class II and class III gynoecia show a more severe response than those of Col WT plants (**Figures 5H,I,K,L**). Class II gynoecia develop the tissue proliferations, and this ectopic tissue was even more evident in the *ahp6* mutant than in WT gynoecia (**Figures 5H,K**). For class III, while WT gynoecia presented the apical–basal defects described in previous reports (reduced, very reduced, asymmetric valves, and at low frequency lack of both valves), most treated *ahp6* gynoecia presented the lack of both valves (**Figure 5L**), indicating an increase in the severity of the response.

We then compared these responses to the responses of *drnl-2* and their respectively WT gynoecia. It became clear that the *ahp6* and *drnl-2* mutants both increased sensitivity or response of early gynoecia to cytokinins. The response of both mutants was severe and produced many gynoecia without valves. However, the effects of these mutants in the response of treated gynoecia at intermediate stages of development (Class II) was opposite, with *ahp6* increasing and *drnl-2* decreasing sensitivity or response to the treatment. We also noticed another conspicuous difference between *drnl-2* and the other genotypes (L*er* WT, Col WT, and *ahp6*): the three phenotype classes developed in chronological order and were easily identified in L*er* WT, Col WT, and *ahp6* gynoecia. The response per developmental stage was clear and generally uniform in these genotypes (Supplementary Figures 5A,C,D). However, the youngest gynoecia of three out of five treated *drnl-2* inflorescences presented a mix of irregular phenotypes that did not follow any chronological or other evident order. These gynoecia presented phenotypic defects that ranged from mild and severe apical–basal defects (observed as the total lack of valves; Supplementary Figure 5B).

In addition, a fourth phenotype of misshaped gynoecia, where the distinct tissues could not be clearly distinguished, was observed in some *drnl-2* gynoecia, not present in the other genotypes or untreated gynoecia. These organs were not able to develop properly and the gynoecium structure was lost (Supplementary Figure 5B; arrowhead). This added further evidence that indicates that *drnl-2* gynoecia have increased cytokinin sensitivity or response at early stages of development.

## Discussion

The loss of *DRNL* function severely affects the development of sepals, petals and stamens, as initially described by Nag et al. (2007). Some defects have also been observed in *drnl-2* gynoecia and fruits, such as valve asymmetry, and more severe defects have been reported in combination with other mutations (Nag et al., 2007; Chandler et al., 2011b; Eklund et al., 2011; Chandler and Werr, 2017). *DRNL* is expressed early during organogenesis. Its expression has been observed before a primordium is histologically visible, and for this reason Chandler et al. (2011b) have proposed it as a founder cell marker of floral organs. Most *drnl-2* gynoecia do not develop into fruits, and the most frequent defect in *drnl-2* gynoecia and fruits was the one-valve phenotype, in up to 30% of mutant gynoecia. This “one valve” may sometimes comprise the region where two normal valves would be present. The replum and valve margin that normally develop between each valve could only be detected on one side of these developing fruits. We found also that, beyond the founder cells and gynoecium primordium, *DRNL* is also expressed at later stages during gynoecium development after the carpel primordia have been specified, and its expression gets confined to the presumptive valves as development progressed. The “one valve” mutant phenotype and *drnl-2* expression in the lateral domain is interesting considering that the different regions of the ovary of the gynoecium are thought to be molecularly similar to the shoot apical meristem (SAM) and lateral organs (leaves) emerging from it (Bowman et al., 1999; Balanza et al., 2006; Ostergaard, 2009). The medial domain, where the CMM and later reproductive tissues develop (Reyes-Olalde et al., 2013), has been compared to the meristematic region, while the valves (lateral domain), have been compared to the lateral organs that initiate from the SAM.

The DRNL function has been associated with lateral organ formation and impaired function causes organ fusion (at low penetrance) in cotyledons and stamens, possibly through *CUC (CUP SHAPED COTYLEDON)* genes (Ikeda et al., 2006; Chandler et al., 2007, 2011b; Nag et al., 2007). Therefore, this particular “one valve” *drnl-2* phenotype may be due to a partial or total fusion of the valves (Supplementary Figure 1), and might further reflect the resemblance between valves and lateral organs. Another possibility, that we cannot discard for the moment, is that some of those gynoecia arose from a single primordium, which then gave rise to a single valve with its own “medial region” on one side. It will be very interesting to test further this possibility.

Some examples of other genes expressed in the presumptive valve region in the developing gynoecium include, among others, *JAGGED, NUBBIN*, and members of the YABBY, KANADI, and HD-ZIP III families. They have been reported to be related to polarity or organ growth and most of their mutants present phenotypes that are different to the ones observed in *drnl-2* (reviewed and reported in: Bowman et al., 1999; Kerstetter et al., 2001; Otsuga et al., 2001; Alvarez and Smyth, 2002; Dinneny et al., 2004, 2006; Roeder and Yanofsky, 2006; Sundberg and Ferrandiz, 2009; Nole-Wilson et al., 2010). Rather, the *drnl-2* phenotypes resemble more those caused by hormonal alterations, and at the functional level, this transcription factor has been suggested to be related to hormonal pathways (Ikeda et al., 2006; Marsch-Martinez et al., 2006; Chandler et al., 2009, 2011a). An indirect connection between DRNL and the auxin biosynthesis pathway through *STYLISH* activation has also been reported (Eklund et al., 2011). Moreover, coincidences between auxin maxima and organ initiation regions in the meristem periphery (Reinhardt et al., 2003; Heisler et al., 2005) have been observed. In the floral meristem, it has been suggested that *DRNL* expression precedes auxin response maxima in the floral organ founder cells, and that it acts synergistically with local auxin biosynthesis and polar transport (Chandler et al., 2011b; Chandler and Werr, 2014). In the gynoecium, auxin response marker expression can be also detected as two foci, suggested to mark the two carpel primordia (Larsson et al., 2014), and this expression has been also observed in *DRNL* marker lines (Chandler et al., 2011b). Mutants affected in auxin biosynthesis, transport, signaling or response (such as multiple *yucca* or *ettin* mutants) or plants treated with auxin transport inhibitors do not develop lateral organs or develop them with severe defects (e.g., Nemhauser et al., 2000; Reinhardt et al., 2000, 2003; Heisler et al., 2005; Cheng et al., 2006). In the Arabidopsis gynoecium, these defects include the alteration of the apical–basal axis, such as valve asymmetry and reduction in valve number (Nemhauser et al., 2000; Cheng et al., 2006; Sohlberg et al., 2006), also observed in *drnl-2* gynoecia. However, it has also been suggested that auxin function, as revealed by the auxin response marker, may be independent of DRNL during early floral development, because its expression is not affected in *drnl-2* (Chandler et al., 2011b).

Interestingly, cytokinin application to developing WT gynoecia can also produce apical–basal defects (Zuñiga-Mayo et al., 2014). Moreover, altered expression of cytokinin pathway genes in mutant or altered *DRNL* backgrounds has been found in global expression analyses (Ikeda et al., 2006; Marsch-Martinez et al., 2006). One of these genes is *AHP6*, reported as a possible target of DRNL (Ikeda et al., 2006). AHP6 is an important element for the correct emergence of organ primordia and the correct distribution of the primordia in the SAM (Besnard et al., 2014). Interestingly, we observed *AHP6* expression at two lateral foci in the pre-patterned incipient carpel primordia (Supplementary Figure 2C), a similar pattern as the one described for *DRNL* (Chandler et al., 2011b). The similarity of expression patterns continued through gynoecium development (**Figure 3** and Supplementary Figure 2), and *AHP6* expression was clearly increased in *DNRL* gain of function backgrounds (**Figure 4**). However, in inflorescences of the loss of function mutant *drnl-2*, we could not detect a clear decrease in *AHP6* expression by qRT-PCR, contrary to what we had expected. This may be because the regulation of *AHP6* by DRNL is tissue-specific, restricted to very narrow domains, and the RNA used for this analysis was isolated from inflorescences and not from isolated gynoecium tissues. Another possible explanation is that *drnl-2* is not a null allele (Nag et al., 2007). It is also possible that we did not detect a strong reduction in *AHP6* expression in the loss of function background because *AHP6* is being regulated by other transcription factors such as MONOPTEROS/AUXIN RESPONSE FACTOR 5 (MP/ARF5; Bishopp et al., 2011; Besnard et al., 2014). However, we confirmed that the increased expression and inducible activation of DRNL was able to increase the expression of *AHP6* in the gynoecium, possibly reflecting what occurs in the lateral domain, and maybe during the earliest stages of organ formation. Based on this, we expected an increase in the sensitivity to exogenously applied cytokinins in *drnl-2* gynoecia. Indeed, we found that young gynoecia showed greater sensitivity to exogenously applied cytokinins. However, we also observed that gynoecia at later developmental stages appeared to be less sensitive to this hormone. The increase in sensitivity in *drnl-2* young organs was revealed by more severe apical–basal defects in comparison to WT. Even, in many *drnl-2* gynoecia valves were not observed. This type of response was similar to that observed in *ahp6* gynoecia. Interestingly, some very young *drnl-2* gynoecia failed to develop and became amorphous organs, which indicates that the lack of *DRNL* function in these developing primordia rendered them unable to counterbalance the excess of cytokinin. The fact that the younger gynoecia are more responsive to cytokinins could be explained through DRNL regulation of *AHP6*, and possibly also *CYTOKININ OXIDASE 7* (*CKX7*), an enzyme that inactivates cytokinins, also proposed to be a direct target of DRNL (Ikeda et al., 2006). The decrease in sensitivity in gynoecia at later stages (as interpreted from their position in the inflorescence stem), was observed as the lack of proliferating tissue that grows from the medial domain of developing gynoecia, normally observed in WT genotypes but not in *drnl-2*. Therefore, the response of *drnl-2* gynoecia to cytokinins appears to be dependent on the stage of development, and suggests that this gene plays a dual function at different stages of development.

The lower sensitivity to cytokinins in more developed gynoecia suggests that *DRNL* is required for the normal response of the tissue to exogenous cytokinins. It could be that the gene is required for the response downstream of cytokinin signaling, or that it modulates cytokinin homeostasis or the sensitivity of the tissue to this hormone, either by regulating the same genes that it regulates at early stages, but in an opposite direction (e.g., repressing instead of activating), or regulating other genes. For example, it has been reported that increased expression of *DRNL/ESR2* caused decreased expression of some type A ARRs (Ikeda et al., 2006), which negatively regulate cytokinin signaling (Hwang et al., 2002; reviewed by Schaller et al., 2015). Also, very recently a mutant in tomato where no leaves develop has been reported (Capua and Eshed, 2017). The authors found that the mutation is located in the ortholog gene of *DRNL* that was called *LEAFLESS (LFS).* Moreover, in *lfs* mutant plants, the development of leaf primordia is not recovered through auxin micro application nor by the expression of *LFS* under the *DR5* promoter. The authors suggest *LFS* might be also regulating cytokinin homeostasis, because genes that participate in the cytokinin pathway (such as type A ARRs and CKXs) were found to be altered in the mutant in global expression analyses (Capua and Eshed, 2017).

Finally, another interesting observation was the asynchronous response of young *drnl-2* gynoecia, in comparison to the rest of evaluated genotypes. This response may be reflecting that the loss of *DRNL* function is not only affecting the morphology of floral organs but also affects the organ spatio-temporal order of initiation or development in the floral meristem. The exacerbated asynchrony observed in treated *drnl-2*, and not detected in *ahp6* mutants, further suggests that DRNL regulates more elements in the cytokinin pathway. Therefore, it might be that Arabidopsis *DRNL*, and possibly the tomato *LFS*, have functions in modulating the cytokinin pathway, or the response to it, through differential gene regulation at different stages of development. **Figure 6** shows a model of the participation of DRNL as a modulator of cytokinin homeostasis and response during gynoecium development, playing at least two different roles as gynoecium development progresses.

## Conclusion

Besides being expressed at the gynoecium initiation stage, *DRNL* participates at further stages during gynoecium development. It differentially modulates the response of the gynoecium to cytokinins at distinct stages, having possibly a dual role during development. It would be interesting to test whether this is the case for other plant species and organs, and to further clarify the mechanisms through which this modulation is achieved. Moreover, considering that *DRNL* has been previously associated to other hormones and is also modulating cytokinin homeostasis or response, *DRNL* may orchestrate different hormonal pathways during the development of the gynoecium and, possible new organs in general.

## Author Contributions

NM-M conceptualized the research, designed experiments, participated in writing, editing, and revising the manuscript. YD-M conceptualized the research, designed and performed experiments, wrote the original drafts, and prepared figures. JS performed experiments, prepared figures, wrote parts, and revised the manuscript. JIR-O performed experiments. SdF conceptualized the research, designed experiments, participated in writing, editing, and revising the manuscript.

## Conflict of Interest Statement

The authors declare that the research was conducted in the absence of any commercial or financial relationships that could be construed as a potential conflict of interest.
