# Supplementary material for: The AP2/ERF Transcription Factor DRNL Modulates Gynoecium Development and Affects Its Response to Cytokinin
Source: Front Plant Sci. 2017 Oct 26;8:1841. doi: 10.3389/fpls.2017.01841 (PMC5662920; doi:10.3389/fpls.2017.01841)
Supplement: Supplementary file 1 [file Image_1.PDF]

Supplementary Material

The AP2/ERF transcription factor DRNL modulates gynoecium development and affects its response to cytokinin

Durán-Medina, Yolanda, Serwatowska, Joanna, Reyes-Olalde, J. Irepan, de Folter, Stefan, Marsch-Martínez, Nayelli *

*** Correspondence: Nayelli Marsch-Martínez**: nayelli.marsch@cinvestav.mx

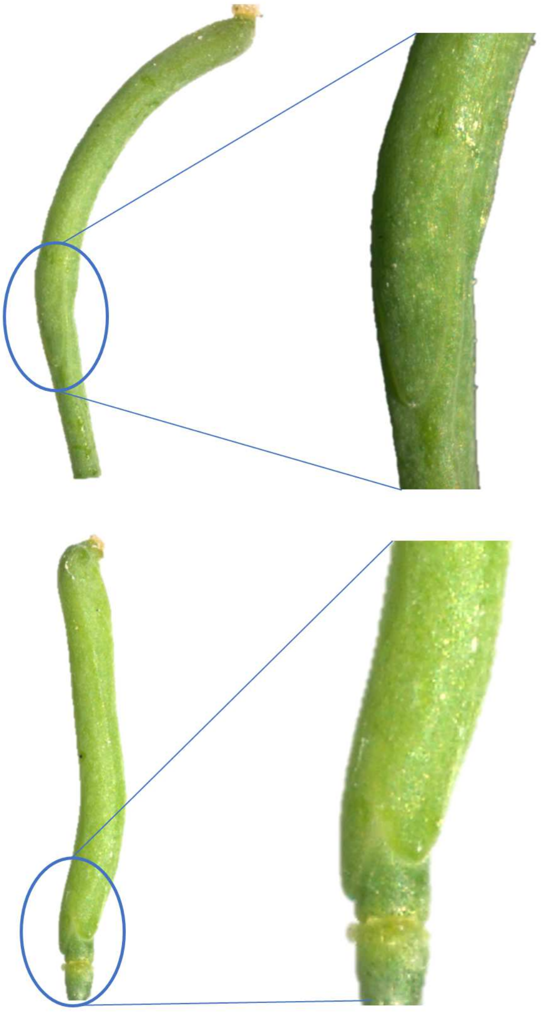

Supplementary Figure 1. Partially fused valves in *drnl-2* gynoecia. The fusion is visible in the basal region of the gynoecium.

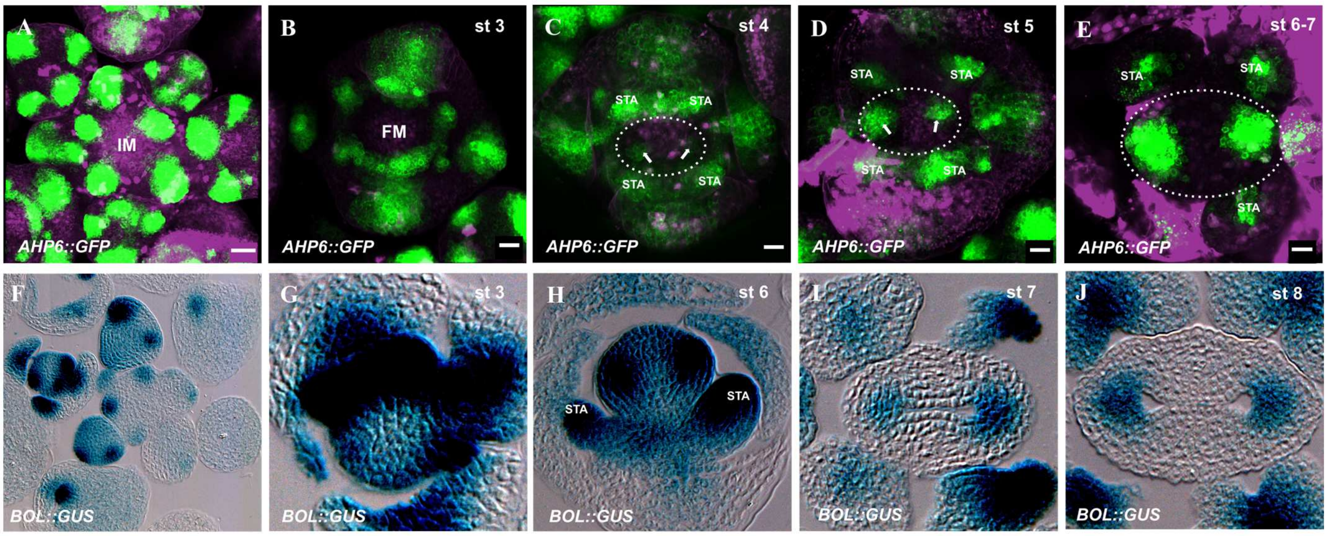

Supplementary Figure 2. Comparison between the expression patterns of *AHP6* (A-E) and *DRNL/BOL* (F-J) during gynoecium development. IM: Inflorescence meristem; FM: Floral meristem; STA: Stamen primordium. Dotted ovals mark developing gynoecia. Arrows point to carpel primordia. Scale bars: 20 µm in A; 10 µm in B-E.

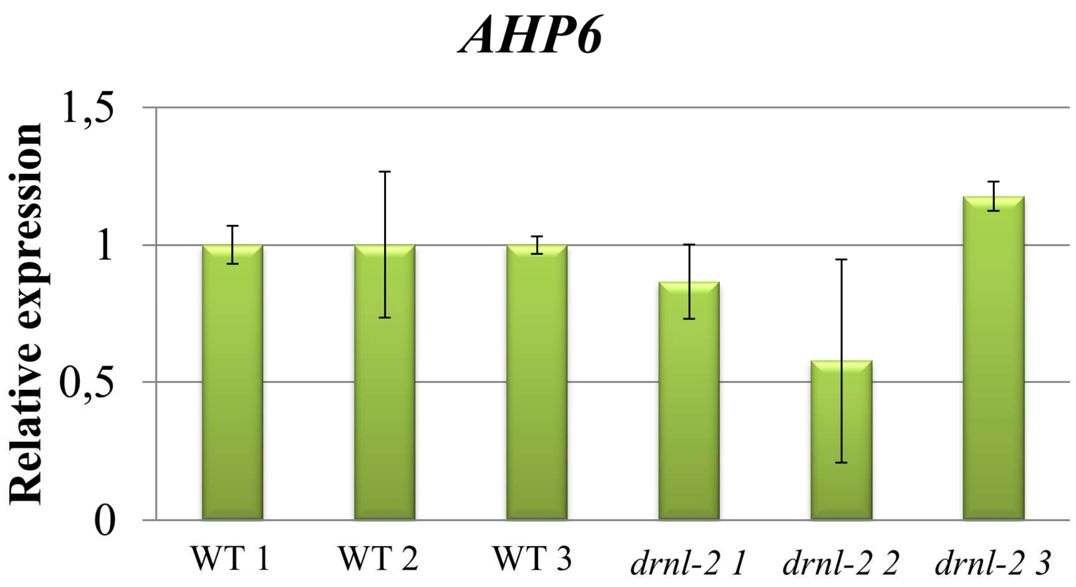

Supplementary Figure 3. *AHP6* relative expression in *drnl-2* inflorescences. Each bar (1, 2 and 3) represents a biological replicate, and standard error bars were calculated from three technical replicates.

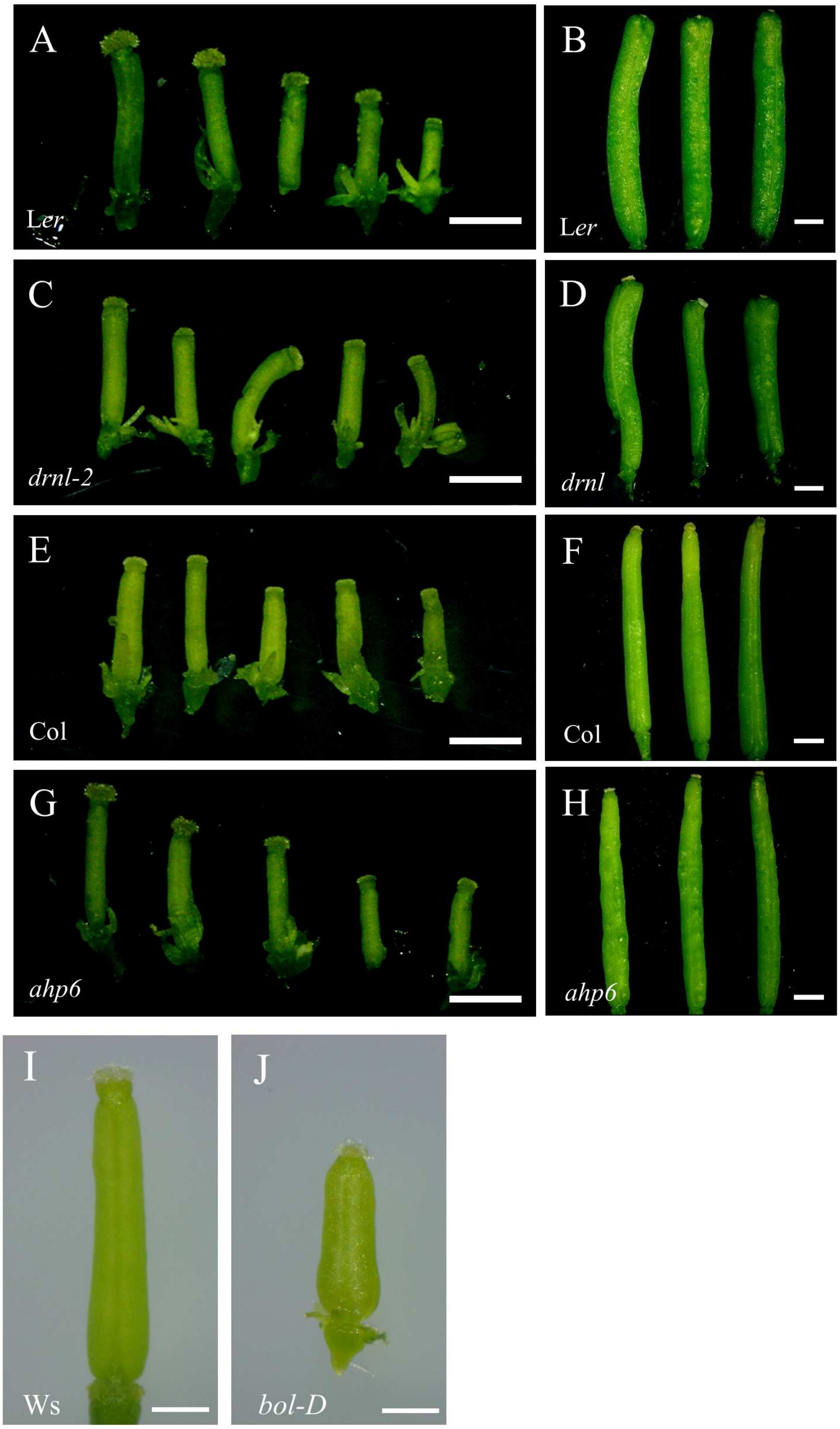

Supplementary Figure 4. Untreated wild type L*er* and Col, and mutant *drnl-2* and *ahp6* pistils and fruits. (A,C,E,G) are pistils, and (B,D,F,H) are fruits. (I,J) are wild type Ws and mutant *bol-D* pistils. Scale bars: 1 mm in A-H; 0.5 mm in I,J.

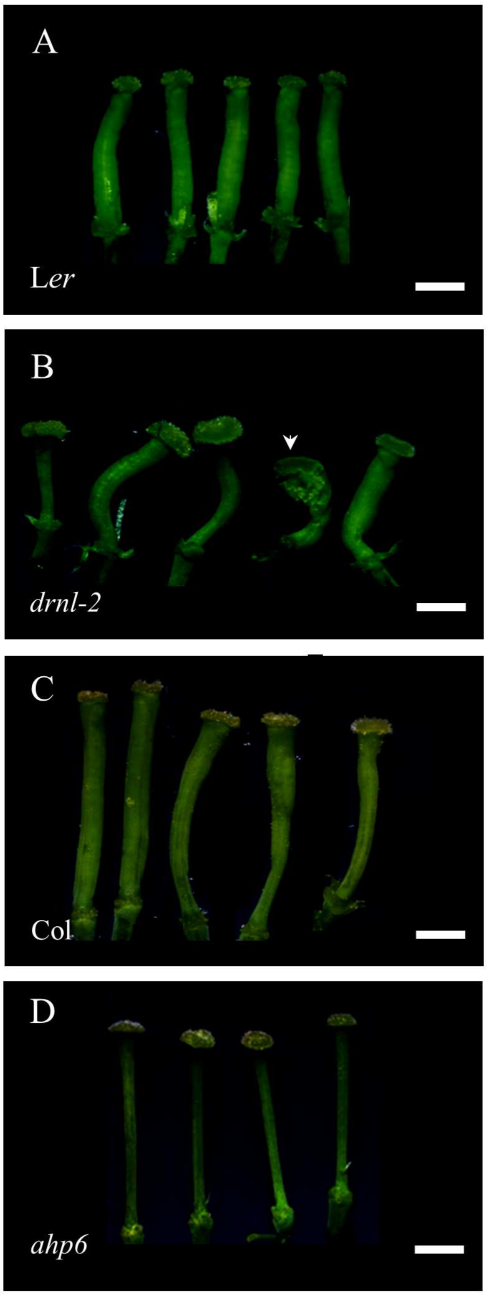

Supplementary Figure 5. Heterogeneous response to cytokinins in *drnl-2* class III gynoecia. (A,C,D) Homogeneous response to cytokinins in treated wild type and *ahp6* gynoecia. (B) Treated *drnl-2* class III gynoecia. The arrowhead highlights a misshapen structure in *drnl-2*. Scale bars: 1 mm in A-D.
